# VHH-based CAR-T cells targeting Claudin 18.2 show high efficacy in pancreatic cancer models

**DOI:** 10.3389/fimmu.2025.1638585

**Published:** 2026-01-02

**Authors:** Ying Xing, Gangqiang Shi, Zhengli Li, Xiancheng Liu, Linghu Nie, Yu Zhang, Yiqin Song, Shilin Sun, Mike K. S. Chan, Michelle B. F. Wong, Krista Casazza, Jonathan R. T. Lakey, Yuping Chen, Xuekai Zhu, Yunfeng Feng

**Affiliations:** 1 Key Laboratory of Clinical Research on Respiratory and Digestive Diseases, Baoding Hospital of Beijing Children’s Hospital Affiliated to Capital Medical University, Baoding, Hebei, China; 2 Research and Development Department, Celest Therapeutics (Shanghai) Co., Ltd, Shanghai, China; 3 European Wellness BioMedical Group, Edenkoben, Germany; 4 Lincoln University College Petaling Jaya, Selangor, Malaysia; 5 European Wellness Academy, Edenkoben, Germany; 6 University of Heidelberg, Heidelberg, Germany; 7 Department of Surgery, University of California, Irvine, Irvine, CA, United States; 8 Institute for Anatomy and Cell Biology, Medical Faculty, University of Heidelberg, Heidelberg, Germany; 9 Department of Biomedical Engineering, University of California, Irvine, Irvine, CA, United States

**Keywords:** VHH, CAR-T cell, CLDN18.2, PDCA, cellular therapy

## Abstract

**Background:**

Pancreatic ductal adenocarcinoma (PDAC) remains one of the deadliest cancers, with a 5-year survival rate below 10%, largely due to late-stage diagnosis and the limited effectiveness of conventional therapies such as surgery, chemotherapy, and radiation. Claudin 18.2 (CLDN18.2)has emerged as a promising target for PDAC. While single-chain variable fragment (scFv)-based CAR-T cells targeting CLDN18.2 have demonstrated therapeutic potential, CAR-T cells engineered with variable heavy-chain-only domains (VHH) exhibit superior efficacy, highlighting the advantages of VHH-based constructs in targeting this antigen. However, the therapeutic efficacy of anti-CLDN18.2 VHH-CAR-T cells remains to be fully elucidated, as previous studies have not comprehensively characterized *in vivo* performance or mechanistic advantages over scFv-based counterparts.

**Methods:**

To characterize the therapeutic potential of anti-CLDN18.2 VHHs, we employed phage display technology to screen a VHH library, resulting in the identification of three positive clones. These candidates were further evaluated and ranked based on binding affinity and multi-round cytotoxicity in Chimeric antigen receptor T (CAR-T) cell models. To reduce immunogenicity, the lead VHH was humanized. VHH-CAR-T cells incorporating this humanized domain were assessed through *in vitro* assays measuring cytokine secretion and target cell lysis, followed by *in vivo* studies to evaluate antitumor efficacy in relevant xenograft models.

**Results:**

High-affinity anti-CLDN18.2 VHHs from phage libraries and engineered CAR-T cells using HM2, a humanized VHH, as the antigen-binding domain were successfully identified. Notably, HM2-CAR-T cells demonstrated potent and sustained cytokine secretion and cytotoxic activity against CLDN18.2-expressing tumor cells *in vitro*. More importantly, these VHH-based CAR-T cells achieved significant antitumor efficacy *in vivo*, underscoring the translational potential of VHH-CAR constructs as a next-generation therapeutic platform with enhanced performance and reduced immunogenicity compared to conventional scFv-based designs.

**Conclusion:**

This study establishes an effective framework for developing CLDN18.2-specific VHHs and demonstrates their successful integration into CAR-T cell therapy. The humanized HM2-CAR-T cells not only maintain high antigen specificity but also exhibit strong effector functions and pronounced antitumor activity in preclinical models. These findings support the clinical promise of VHH-based CAR-T cells as a next-generation immunotherapy for CLDN18.2-expressing malignancies, particularly PDAC, where effective treatment options remain limited.

## Introduction

Pancreatic ductal adenocarcinoma (PDAC) remains one of the deadliest malignancies, with five-year survival rates below 10% and limited effective treatment options (1). Chimeric antigen receptor T (CAR-T) cell therapy has achieved remarkable success in the clinical management of hematologic malignancies (2). CARs are engineered transmembrane proteins introduced into immune effector cells (e.g., T cells, natural killer (NK) cells, macrophages) to enable targeted recognition and destruction of cells expressing specific surface antigens (3). However, its broader application to solid tumors remains hindered by several challenges, particularly the identification and selection of ideal targets (2, 4–6).

Among emerging candidates, Claudin-18.2 (CLDN18.2) has gained attention due to its tumor-restricted expression profile. CLDN18.2, an epithelial tight junction protein aberrantly expressed in a majority of PDAC tumors but restricted in normal tissues, has emerged as an attractive tumor-specific antigen for targeted immunotherapy (7). CLDN18.2 expression becomes aberrant during malignant transformation. Although overexpression of CLDN18.2 has been reported in several cancer types (8–11). PDAC as one of the most aggressive and lethal malignancies, highlights the highly warranted need for the development of CLDN18.2-targeted therapies (7).

Approximately 59.2% of primary PDAC cases exhibit CLDN18.2 positivity, with a predominant 2+ expression level (12). This makes CLDN18.2 an attractive target for CAR-T cell therapy. A favorable safety profile with no significant adverse events, treatment-related mortality or severe neurotoxicity among patients with metastatic adenocarcinoma including gastric cancer (n=7) and pancreatic cancer (n=5), treated with CLDN18.2-targeted CAR-T cells (CT041) was reported (12). The overall response rate (ORR) of 48.6% and disease control rate (DCR) of 73.0%, with a 6-month duration of response rate of 44.8% underscored the clinical potential of CLDN18.2-targeted CAR-T cells. However, while single-chain variable fragment (scFv)-based CAR-T cells against CLDN18.2 have demonstrated initial promise, their large size and complex structure can compromise tumor penetration and increase immunogenicity (13). In contrast, variable heavy-chain-only domains (VHHs) offer superior stability, reduced immunogenicity, and enhanced tissue diffusion (14).

The unique potential of VHH-based CAR-T cell therapy has begun to emerge (15, 16). VHHs, derived from variable domain of heavy chain of heavy chain antibody, are compact antigen-binding fragments (~15kDa), also referred to as Nanobodies (Nbs), that in comparison to traditional antibodies offer distinct advantages including enhanced thermal and chemical stability, as well as the ability to facilitate the assembly of multivalent formats (17, 18). In addition, VHH-based CAR-T cells targeting B7-H3 have been shown to produce higher transduction efficiency and more pronounced tumor regression compared to scFv-based CAR-T cells in pancreatic cancer models15. Despite these advancements, the therapeutic efficacy of VHH-based anti-CLDN18.2 CAR-T cells remain underexplored, necessitating comprehensive experimental validation to assess their functional advantages and translational potential (9). Here, we report a streamlined pipeline for isolating high-affinity anti-CLDN18.2 VHHs via phage display, followed by humanization to minimize host immune responses.

Herein we report our development of effective strategies for identifying VHHs that bind with high affinity and specificity to CLDN18.2 epitope, selected the humanized candidate, HM2 selected for generating CAR-T cells, and the significant cytotoxicity against CLDN18.2 positive tumor cells in HM2-CAR-T cells *in vitro*. We next translated these findings in an immunodeficient mouse model, showing that HM2-based CAR-T cells exhibit notable antitumor activity. The collective *in vitro* and *in vivo* experiments, suggests that HM2-based CAR-T cells as a promising therapeutic agents for human cancers expressing CLDN18.2 such as PDAC.

## Materials and methods

### Cell culture

The 293T cell line was obtained from the American Type Cell Culture Collection (ATCC). NUGC-4 human gastric tumor cells were purchased from Cobioer Biosciences Co., LTD (Nanjing, China). AsPC-1 human pancreatic cancer cells, CHO-K1 Chinese Hamster Ovary cells were provided by the National collection of Authenticated cell Cultures (Shanghai, China). Peripheral blood mononuclear cells (PBMCs) were purchased from Zhejiang Maishun Biotechnology Co., Ltd (Zhoushan, China). 293T cells were used to produce the retrovirus by transfecting the retroviral packaging plasmids into cells. NUGC-4 cells included medium CLDN18.2 positive cells used in the cytotoxicity assays. AsPC-1 and 293 T cells were transduced by retroviruses encoding the CLDN18.2–luciferase to stably express human claudin 18.2 (CLDN18.2-AsPC-1 and CLDN18.2-293T cell) and luciferase for luciferase-based cytotoxicity assay. CHO-K1 cells were transduced by retroviruses encoding the human CLDN 18.1 and CLDN18.2, mouse CLDN 18.2 to stably express human claudin 18.1 (CHO-K1/18.1 cell), human claudin 18.2 (CHO-K1/18.2 cell) and mouse claudin 18.2 (CHO-K1/Mouse18.2 cell), respectively. Each were used for analysis of the binding specificity of VHH. PBMCs were used to isolate human primary T and NK cells. The isolated T and NK cells were transduced by retroviruses encoding the CAR-gene to stably express VHH based CAR (VHH-CAR-T and VHH-CAR NK). The 293T cells were cultured in Dulbecco’s modified Eagle medium (DMEM) (Solarbio) supplemented with 10% fetal bovine serum (FBS) (Gibco), 100 U/ml penicillin and streptomycin sulfate (Gibco). NUGC-4 and AsPC-1 cells were maintained in Roswell Park Memorial Institute (RPMI) 1640 medium (Gibco) supplemented with 10% FBS, 100 U/ml penicillin and streptomycin sulfate. Primary human T cells were cultured in X-VIVO 15 medium (Lonza) supplemented with 10% FBS, 1% penicillin and streptomycin sulfate, and 250 IU/mL recombinant human IL-2. NK cells were cultured in Gibco CTS NK-Xpander medium (Gibco) supplemented with 10% FBS, 1% penicillin and streptomycin sulfate, and 1000 IU/mL recombinant human IL-2.

### Generation of VHH’s

CLDN18.2 VHHs were screened by phage display technology as described in prior studies (15, 19). Briefly, a healthy *alpaca* was immunized with 2× 107 CLDN18.2-expressing 293T cells, with Gerbu adjuvant as an immunopotentiator. A total of five immunizations were performed with an interval of 14 days between each immunization. Next, whole blood from the alpaca was harvested seven days after the final immunization. B cells were isolated from the whole blood. Extracted RNA was used as a template for synthesizing cDNA by reverse transcription (Super Scrip III First Strain, Invitrogen). Synthesized cDNA was used as a template for the polymerase chain reaction (PCR) to amplify the VHH sequences. Amplified VHH fragment products were collected and purified, then ligated to phagemid vector pDisplay (Invitrogen). The ligated products were transformed into an Escherichia coli–competent strain, SS320, by electroporation. Biotinylated human Claudin 18.2 (Cat# CLD-HE1822B, Kactus Biosystems Co., Ltd.) and human Fc proteins were used in the bio-panning to enrich positive phages and eliminate non-specific phage binders. The purity of the selected CLDN18.2 VHHs was confirmed by SDS-PAGE analysis. For the assessment of specific binding of CLDN18.2 VHHs, the VHH antibodies were incubated with the corresponding target cells. Subsequently, binding affinity was evaluated using flow cytometry.

### Humanization of VHH’s

To humanize VHHs, the CDR grafting method was employed (20). First, the VHH sequences were searched against the human germline sequence databases with IMGT/V-QUEST (http://www.imgt.org/IMGT_vquest/input), and the closest heavy chain (VH) of human antibodies were identified. The framework regions of the selected human antibody were used as the template for grafting the CDRs of VHHs to them. This process results in the 100% humanization of VHHs without amino acid manipulations of the native CDRs. To mitigate antigen-binding affinity loss during CDR grafting, various back mutations were designed and further introduced into the fully (100%) humanized VHH to substitute the corresponding residues Germline/template identification. Each camelid VHH sequence was queried against human germline VH databases using IMGT/V-QUEST to identify the closest human VH framework(s) as candidate templates. CDR boundaries were defined according to IMGT numbering.

CDR grafting. The VHH CDRs (IMGT-defined CDR1–3) were grafted onto the chosen human VH frameworks to generate a fully human framework with native VHH antigen-binding loops.

Preservation of VHH-specific framework features. Where necessary to preserve VHH fold stability and solubility, framework residues known to contribute to VHH structural integrity (i.e., residues that substitute for light-chain contacts in camelid VHHs) were retained or selectively returned to the camelid identity in the humanized scaffold. Decisions were made on a residue-by-residue basis guided by sequence alignments and structural considerations.

Back-mutation design and structural assessment. To mitigate potential loss of affinity due to grafting, we identified framework (Vernier-zone) residues likely to influence CDR conformation (by sequence comparison and structural modeling). Targeted back-mutations were introduced, and modeled constructs were evaluated in silico for CDR orientation and steric compatibility. Variants incorporating selected back-mutations were produced and screened.

Functional screening. All humanized variants were expressed and screened for (i) expression and biophysical behavior, (ii) cell-surface binding to CLDN18.2-expressing cells (flow cytometry), and (iii) functional activity when formatted as CARs in the multi-round cytotoxicity assay described in the manuscript. The humanized candidate designated HM2 (derived from clone 2-B08) retained binding and CAR functionality and was selected for downstream studies.

### VHH-CAR construction

For the construction of the CLDN18.2 VHH-CAR, the CLDN18.2 Nb domain was incorporated into the second-generation CAR, comprising a signal peptide, CD8α hinge, CD8 transmembrane segment, OX40 costimulatory domain, and CD3 zeta activation signal. In some experiments, IL15 fusion protein containing IL15 and IL15 receptor alpha was put behind the CAR. Puromycin selection gene was included in all the CAR vectors. CLDN18.2 VHH-CAR constructs were cloned into the pCigar retroviral vector (synthesized by GenScript, China) and validated by sequencing. Retroviral supernatants were generated by co-transfecting 293T cells with the pCigar plasmid and the packaged plasmid pCL-10A1. The retroviral supernatants were harvested 48 hours after transfection.

### VHH-CAR-T cell generation

To generate VHH-CAR-T cells, the primary human T cells were isolated from PBMCs using the CD3 MicroBeads, following the manufacturer’s protocol (Cat# 130-050-101, Miltenyi Biotec). Subsequently, the primary T cells were stimulated with Activation/Expansion CD3/CD28 Beads. The T cells were then cultured in X-VIVO 15 medium, supplemented with 10% FBS, 1% penicillin and streptomycin sulfate, and 250 IU/mL recombinant human IL-2 (Novoprotein, GMP-CD66-1mg). Following 48 hours of activation, the T cells were transduced with a CAR-encoding retrovirus in RetroNectin (Takara)-coated plates and further incubated in the presence of human IL-2 (hIL-2). On Day 11 post-transduction, CAR expression was assessed by flow cytometry.

### VHH-CAR NK cells generation

To prepare VHH-CAR NK cells, the remaining PBMCs after depletion of T cells using the CD3 MicroBeads were used to produce NK cells, which were stimulated with K562-based feeder cells (21, 22). The NK cells were cultured in Gibco CTS NK-Xpander medium, supplemented with 10% FBS, 1% penicillin and streptomycin sulfate, and 1000 IU/mL recombinant human IL-2 (Novoprotein, GMP-CD66-1mg). After five days of activation, the NK cells were transduced with a CAR encoding retrovirus in RetroNectin (Takara)-coated plates, followed by continued incubation in the presence of hIL-2. At 72h after transduction, CAR expression was assessed by flow cytometric analysis.

### Short term *in vitro* cytotoxicity and multi-round cytotoxicity assay

The cytotoxicity of CLDN18.2 VHH-CAR-T or VHH-CAR NK cells *in vitro* was analyzed with a luciferase-based assay. Briefly, target tumor cells were seeded overnight in a 96-well plate at a concentration of 5 × 10^4^ cells/well. All target cells were engineered to express luciferase (Luc). Then, VHH-CAR-T cells were added to the plate at effector-to-target ratios of 1:1, 1:3, 1:4 and 1:9 and VHH-CAR NK cells were added to the plate at effector-to-target ratios of 1:1, 1:4, 1:16 and 1:64, respectively. Twenty hours after co-culture, the luciferase activity was analyzed using the GMone-step Luciferase Reporter Gene Assay Kit (Genomeditech). In the multi-round cytotoxicity assay, effector cells were co-cultured with target cells at an E/T ratio of 1:1, The luciferase activity was analyzed at the end of each round of co-culture. In the second and the following round, 5 × 10^4^ fresh CLDN18.2-AsPC-1 or NUGC4 cells were added into all wells co-cultured with the remaining effector cells for another 48 h.

### Cytokine release assay

A cytokine release assay was performed by co-culture of tumor cells with transduced T cells at an effector-to-target ratio of 1:1 for a duration of 18 h. Subsequently, the supernatant was collected for the analysis of cytokine secretion. The levels of interferon (IFN), tumor necrosis factor (TNF), and granulocyte-macrophage colony-stimulating factor (GM-CSF) in the culture supernatants were quantified using enzyme-linked immunosorbent assay (ELISA).

### Flow cytometry

For the assessment of CLDN18.2 expression on the cell surface, tumor cells were detected using a rabbit anti-human Claudin 18.2 monoclonal antibody (mAb) conjugated with allophycocyanin (APC), applied at a dilution of 1:100 (ABclonal, catalog number A23392). To evaluate the efficiency of retroviral transduction, CAR expression on T or NK cells was detected using a rabbit anti-Camelid VHH cocktail antibody conjugated with phycoerythrin (PE) (GenScript, catalog number A02018-200).

### *In vivo* validation studies

The animal experiments were conducted in compliance with the approval of the Qishang Ethics Committee on Animal Care. In the current study, the humane endpoints were strictly defined. Mice were euthanized if 1) the tumor volume exceeded 2,000 mm^3^, or 2) the mice lost more than 20% of their body weight during the treatment period. NOD scid gamma (NPSG) mice, which are immunodeficient mice, were purchased from Phenotek Biotechnology (Shanghai) Co., Ltd. For the establishment of tumor-bearing mouse models, 6- to 8-week-old NPSG mice were subcutaneously injected with either 3 × 10^6^ CLDN18.2-AsPC-1 tumor cells. At eight days post-inoculation, the mice received a tail vein injection of 1 × 10^7^ or 3 × 10^6^ CAR-T cells. Throughout the experimental period, NPSG mice were closely monitored for any physical symptoms. Tumor volumes were estimated using the formula: 1/2 × length × width². Additionally, the survival rate of the mice was assessed, and their body weight was tracked at regular intervals.

Mice were humanely euthanized via carbon dioxide (CO2) asphyxiation upon meeting one of the following humane endpoints: 1) tumor volume exceeding 2000mm3; or 2) loss of more than 20% of initial body weight during the treatment period, Once euthanasia criteria were satisfies, each mouse was placed in a transparent induction chamber. Pure CO2 was then introduced at a flow rate displacing 30% of the chamber volume per minute. After loss of consciousness was confirmed CO2 administration was continued for an additional three minutes to ensure euthanasia.

### Statistical analysis

Statistical analyses were performed in GraphPad Prism 6. Data are presented as mean ± SD, unless otherwise specified. Statistically significant differences are indicated in each figure and/or figure legend. A p-value of less than 0.05 was considered statistically significant.

## Results

### Identification of VHHs specifically binding to CLDN18.2-expressing cells

**Figure 1:**
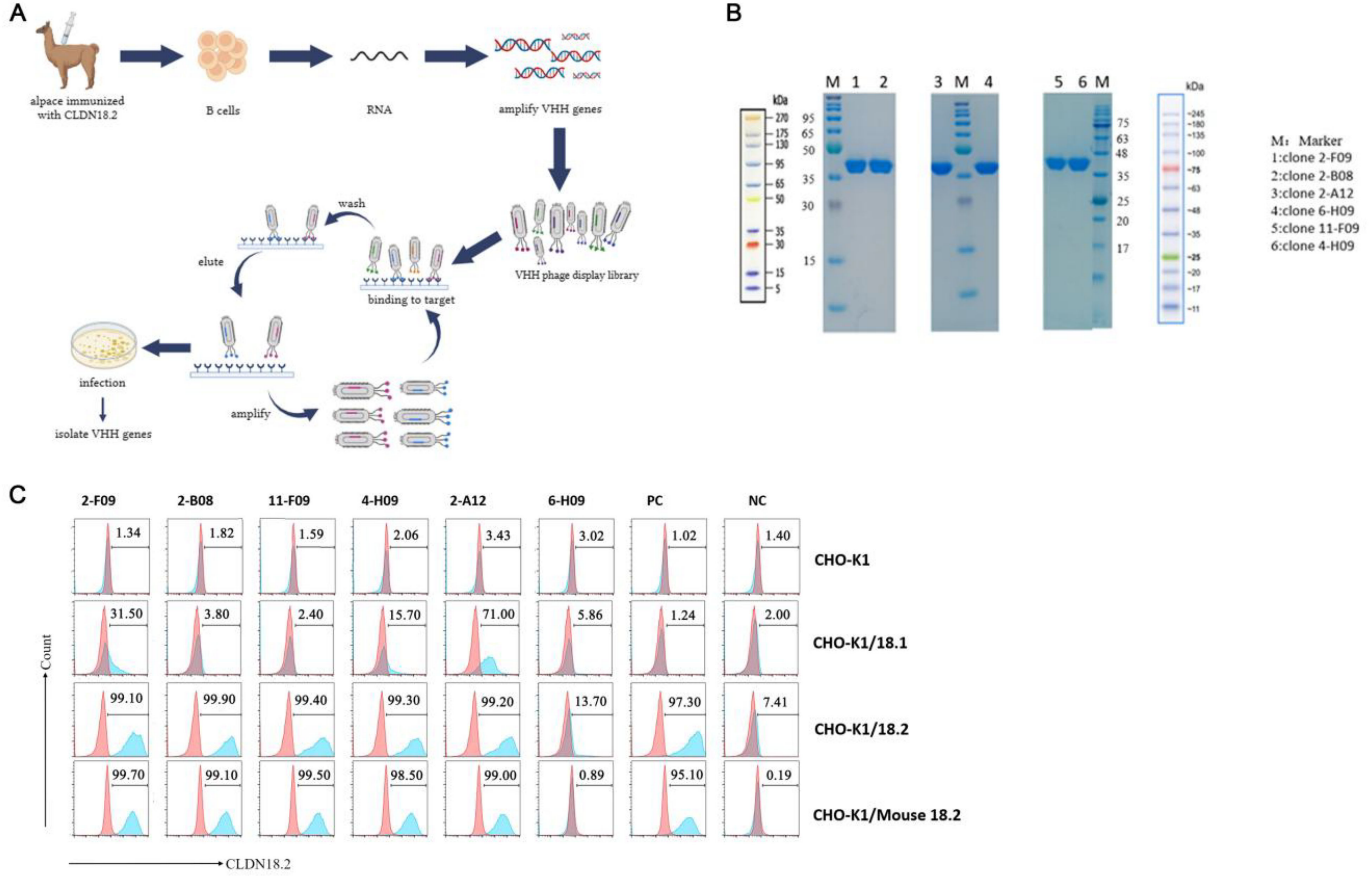
Identifying VHHs that specifically bind to CLDN18.2-expressing cells. **(A)** Schematic representation shows phage panning. The anti-claudin 18.2 phage binders were isolated from 4 rounds of panning of Alpaca phage libraries. After panning and selection of colonies with high affinity, the variable fragment antibody (VHH) genes were sequenced. **(B)** SDS-PAGE analysis of VHH-Fc fusion proteins, which consists of the VHH and the human immunoglobulin heavy constant gamma 1 (IGHG1) residues 104-330, with mutations D239E and L241M. **(C)** Flow cytometry was performed to determine the specificity of 2-B08, 2-F09, 11-F09, 4-H09, 2-A12, 6-H09. Claudin18.1, Claudin18.2, Mouse Claudin18.2 were expressed on the surface of CHO-K1/18.1, CHO-K1/18.2, CHO-K1/Mouse 18.2, respectively. PC and NC represent positive control and negative control, respectively.

To generate CLDN18.2 VHHs with high affinity and specificity, an alpaca was immunized with CLDN18.2-expressing 293T cells. **Figure 1a** presents B cells from immunized alpaca isolated to construct the VHH library by amplifying VHH genes (**Figure 1A**). Subsequently, a phage display library (approximately 1.04 × 10^9^ plaque-forming units) was created. To isolate CLDN18.2-specific VHHs, biotinylated human Claudin 18.2 and human Fc proteins were employed for enriching positive antibodies and depleting non-specific clones, respectively. Finally, four rounds of biopanning were conducted. Phages displaying VHHs with specificity for human CLDN18.2 were effectively enriched. Furthermore, the VHHs were fused with the human immunoglobulin heavy constant gamma 1 (IGHG1) residues 104-330, with mutations D239E and L241M. After expression, the VHHs existed as dimers. Six VHH candidates were identified and expressed, and purified proteins were detected by sodium dodecyl sulfate polyacrylamide gel electrophoresis (SDS–PAGE), showing bands consistent with the expected molecular size of 38 kDa (**Figure 1B**).

Flow cytometry analysis revealed that VHHs 2-B08 and 11-F09 specifically recognized human CLDN18.2 overexpressing cells, but not human CLDN18.1 overexpressing cells. Additionally, VHHs 2-F09, 4-H09, and 2-A12 appeared to recognize both human and mouse CLDN18.2, as well as human CLDN18.1. In contrast, 4-H09 appeared to be selective for human and mouse CLDN18.2. VHH 6-H09 showed very weak binding to both human CLDN18.2 and CLDN18.1 (**Figure 1C**). These findings confirmed successful identification of VHHs (2-B08, 11-F09, and 4-H09) with targeted specificity for CLDN18.2.

### Potent humanized VHHs are identified based on affinity and CAR-T function evaluation

**Figure 2:**
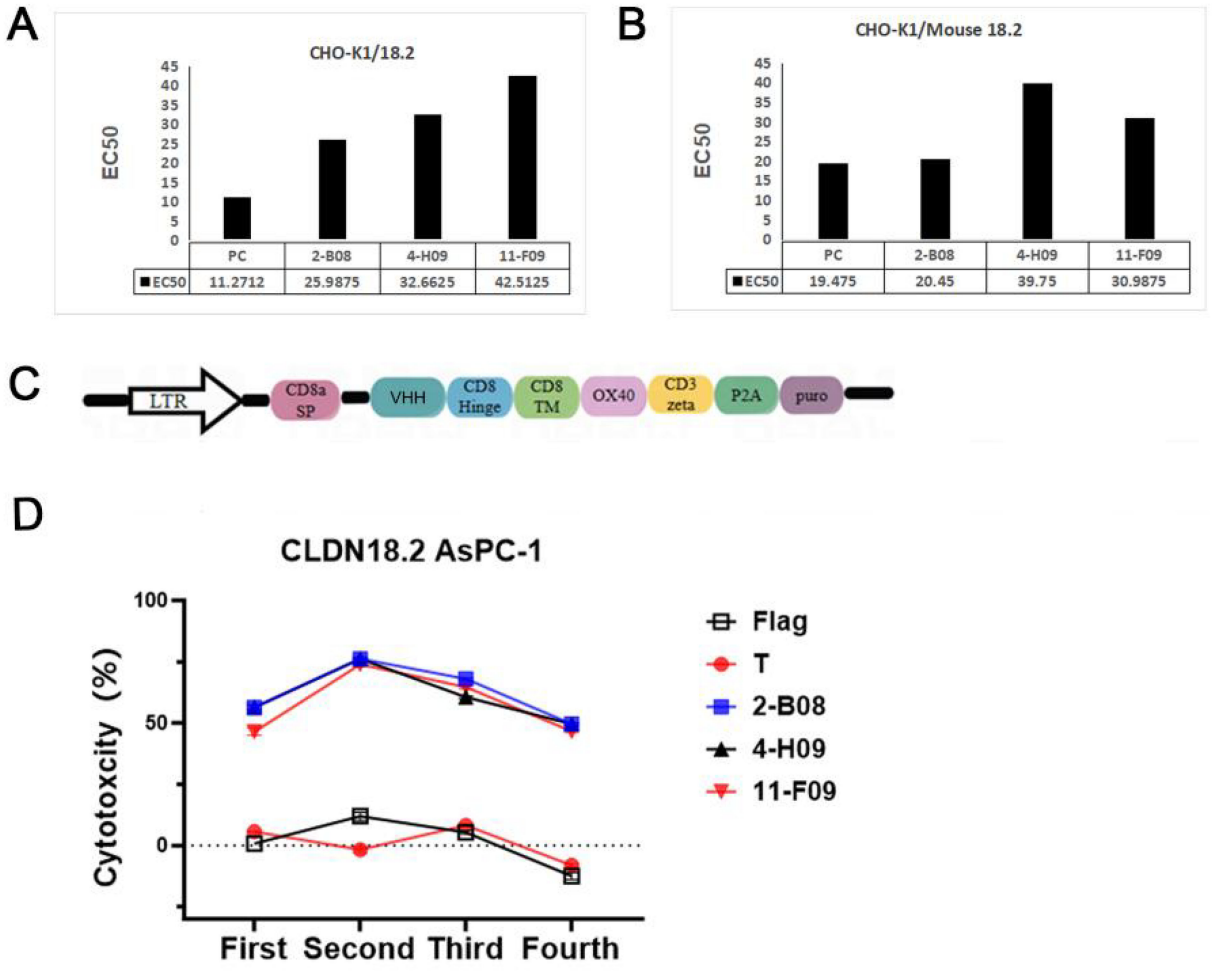
VHHs with high affinity to CLDN18.2-expressing cells and strong long-term cytotoxicity *in vitro* are selected for humanization. **(A)** The affinity of 2-B08, 11-F09, 4-H09, PC (positive control) to CHO-K1/18.2 cells. The EC50 (concentration for 50% of maximal effect) is shown below the column graph. **(B)** The affinity of 2-B08, 11-F09, 4-H09, PC to CHO-K1/Mouse 18.2 cells. The EC50 is shown below the column graph. **(C)** Schematic of retroviral vector constructs encoding VHH for transduction into T cells. **(D)** The multi-round cytotoxicity assay of T cells expressing the indicated positive clone-based CAR.

**Figure 3:**
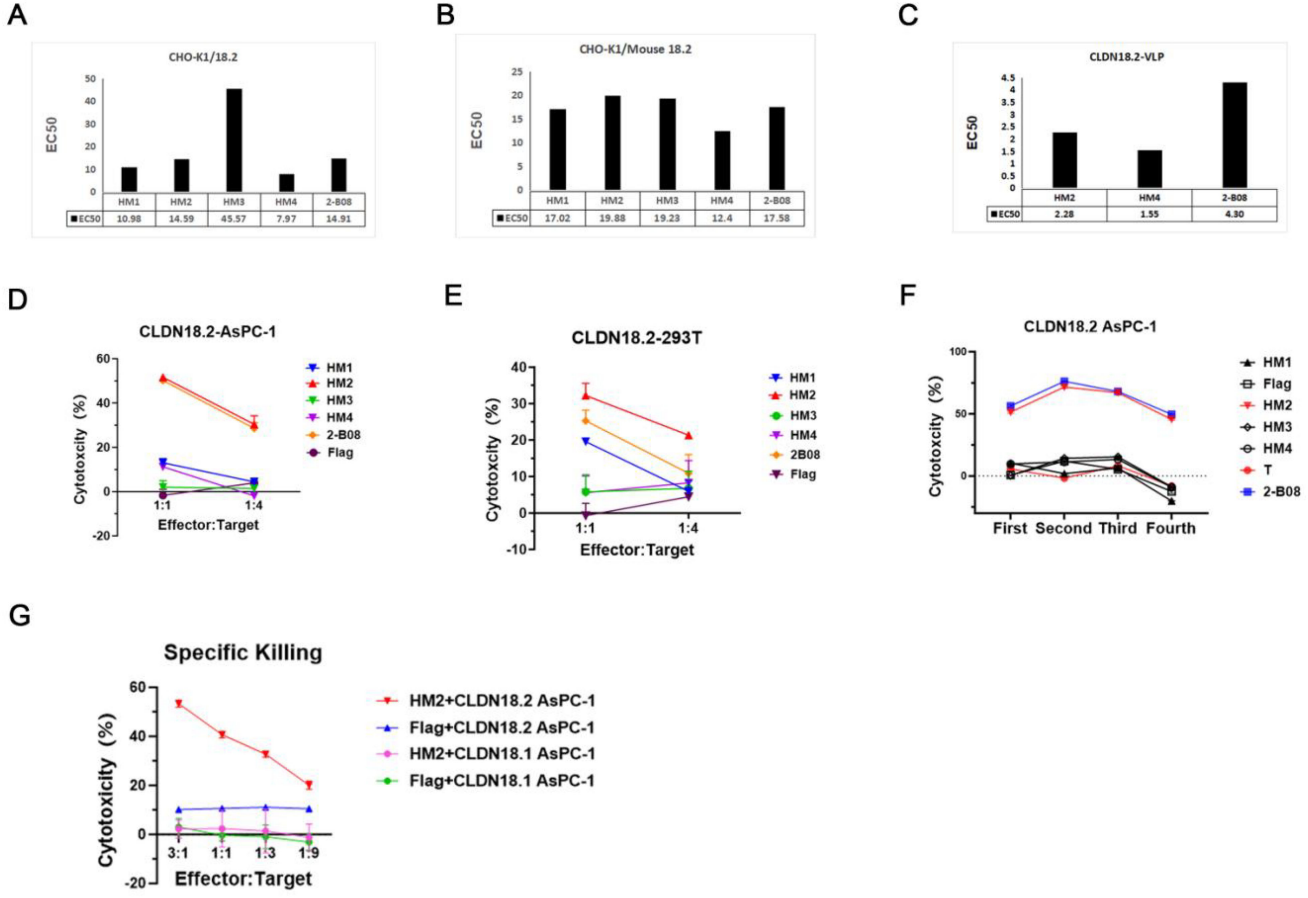
Potent humanized VHH was chosen based on affinity and CAR-T function evaluation. **(A)** The affinity of HM1, HM2, HM3, HM4, 2-B08 to CHO-K1/18.2 cells. The EC50 is shown below the column graph. **(B)** The affinity of HM1, HM2, HM3, HM4, 2-B08 to CHO-K1/Mouse 18.2 cells. The EC50 is shown below the column graph. **(C)** The affinity of HM2, HM4, 2-B08 to claudin18.2-protein. The EC50 is shown below the column graph. CLDN18.2-VLP means CLDN18.2 full length protein. **(D, E)***in vitro* 20h cytotoxicity assays of VHH-CAR-T cell against CLDN18.2-AsPC-1 and CLDN18.2-293T cells, respectively. **(F)** The four-round cytotoxicity assay of VHH-CAR-T cells against with CLDN18.2-AsPC-1. **(G)** Cytotoxicity assay of HM2-CAR-T cells against CLDN18.1-expressing cells.

Binding affinity is crucial for the therapeutic efficacy of VHHs and their application in CAR-T or CAR-NK cells (23). To determine the optimal VHH for CAR construction, the binding affinity of the candidate VHHs was assessed using fluorescence-activated cell sorting (FACS). A curve was plotted with the molar concentration of the antibody on the x-axis and the Mean Fluorescence Intensity (MFI) value on the y-axis. As shown in **Figures 2A, B**, EC50 of VHHs binding to CHO-K1/CLDN18.2 and CHO-K1/Mouse CLDN18.2 were 25.98-32.66 nm and 20.45-39.75 nM, respectively. 2-B08 had the lowest EC50 values with CHO-K1/CLDN18.2 and CHO-K1/Mouse CLDN18.2, indicating that 2-B08 had the highest binding affinity for CLDN 18.2 overexpressing cells. To verify the performance of the three VHHs in CAR-T cells, second-generation CAR vectors containing different VHHs were constructed, and CAR-T cells were successfully produced. The multi-round cytotoxicity assay demonstrated that 2-B08 CAR-T cells maintained long-term cytotoxicity, comparable to 11-F09 or 4-H09 based CAR-T cells (**Figure 2C**). Additionally, based on the sequence homology analysis between VHHs and the framework region of human VH, the homology percentages for 2-B08, 11-F09, and 4-H09 were 75.5%, 72.9%, and 69.5%, respectively. The VHH 2-B08 showed the highest homology with the framework region of human VH. In addition to evaluating the affinity of the VHHs for 18.2-expressing cells, we conducted a comprehensive assessment of their cytotoxic potential as CARs using a multi-round cytotoxicity assay. Based on these results, we selected 2-B08 for humanization, as shown in **Figure 2**. Consequently, 2-B08 was selected for further humanization to mitigate immunogenicity. After the humanization designs were completed, four humanized VHH clones were selected for further analysis. To analyze the affinity of humanized VHHs (HM variants), the FACS (fluorescence-activated cell sorting) assay was performed. In the FACS analysis, the live cell population were gated based on the Forward Scatter (FSC) and Side Scatter (SSC) parameters to determine the proportion of live cells. The updated data can now be found in **Supplementary Figure S2A**. While SPR/BLI are the accepted gold standards for deriving kinetic constants (k_on, k_off) and an absolute KD for antibody–antigen interactions several orthogonal strategies intended to enable SPR/BLI measurements were conducted The HMs maintained high binding affinity to CLDN18.2 expressing cells, with EC50 value ranging from 7.97-45.57nM and 12.4-19.88nM for CHO-K1/CLDN18.2 and CHO-K1/Mouse CLDN18.2 cells, respectively (**Figures 3A, B**). In addition, the EC50 values for humanized VHHs binding to CLDN18.2 full length protein (CLDN18.2-VLP, cat# CL2-H52P7, ACRO biosystems) ranged from 1.55 to 4.3nM (**Figure 3C**). The values were comparable to that of 2-B08, indicating that humanization did not significantly affect the affinity of 2-B08. Further, we conducted *in vitro* 20h cytotoxicity and a multi-round cytotoxicity assay to compare the cytolytic capacity of CAR-T cells expressing different constructs. HM2-CAR-T cells exhibited a stronger cytotoxicity against CLDN18.2-AsPCP-1 cells compared to other humanized VHH CAR-T cells (**Figures 3D, F**), indicating HM2 was the most potent humanized VHH for engineering T cells to lyse CLDN18.2-positive tumor cells. CLDN18.2-expressing 293T as target cells yielded similar results. To further determine the specificity of HM2, we conducted killing experiments using CLDN18.1-expressing AsPC-1 as target cells and Membrane Proteome Array (MPA) analysis. The cytotoxicity assay confirmed that HM2-CAR-T cells specifically lysed CLDN18.2 positive cells without affecting CLDN18.1-expressing cells (**Figure 3G**). Additionally, the MPA analysis against 5886 human membrane proteins further confirmed no off-target binding (**Supplementary Figure S3**).

### HM2-CAR-T cells exhibit a significant effector function *in vitro*

**Figure 4:**
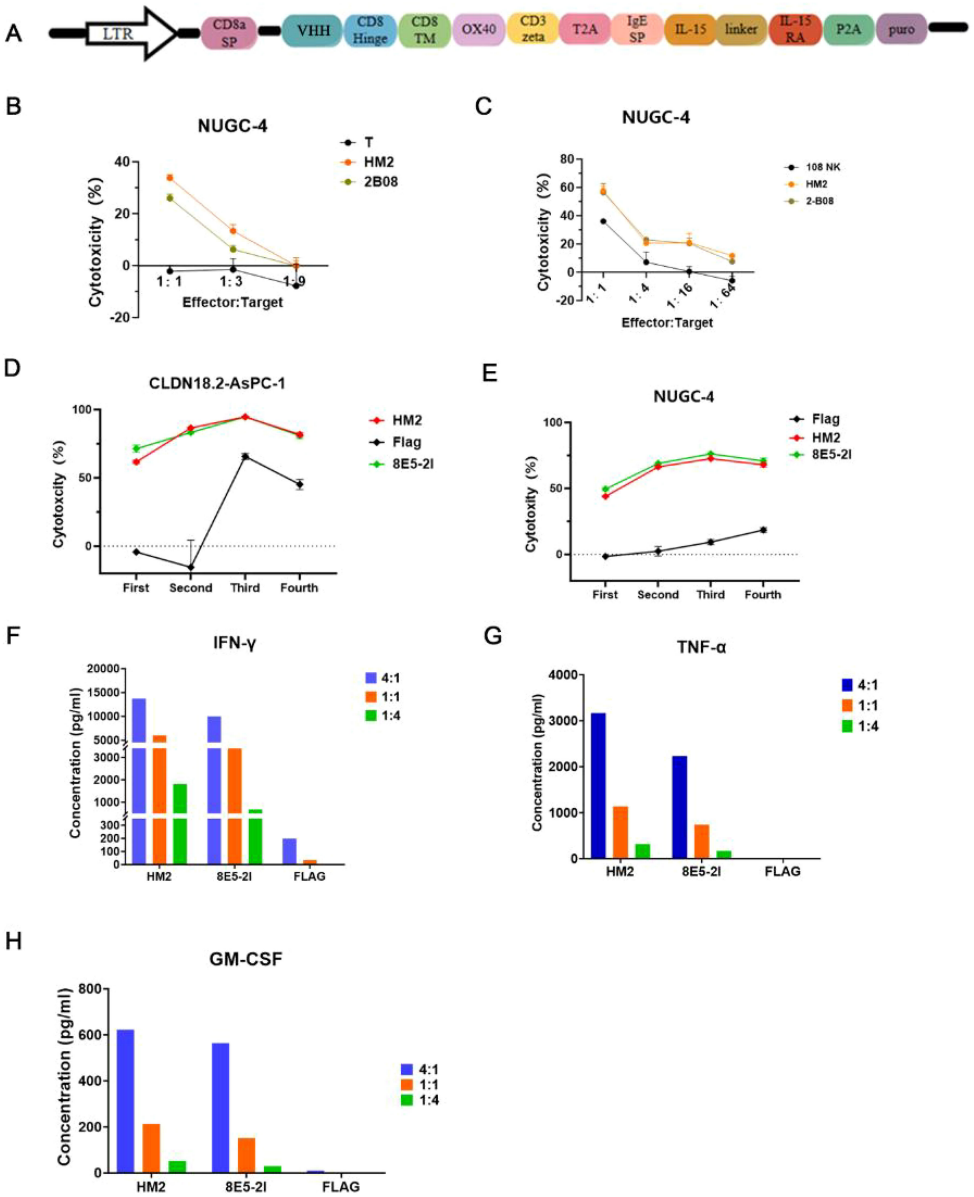
HM2-CAR cells exert an effector function *in vitro*. **(A)** Schematic of retroviral CAR constructs encoding VHH for transduction into NK cells. **(B)***in vitro* cytotoxicity assays of HM2-CAR-T cells against NUGC-4 cells. The representative data are shown, and experiments were replicated with more than three donors. **(C)***in vitro* cytotoxicity assays of HM2-CAR NK cells against NUGC-4 cells. The "108 NK" refers to NK cells isolated from donor 108. The representative data are shown, and experiments were replicated with more than three donors. **(D, E)** The multi-round cytotoxicity assay of HM2-CAR-T cells against CLDN18.2-AsPC-1 and NUGC-4 cells, respectively. **(F-H)** CAR-T cells were cocultured with target cells and IFN-γ, TNF-α, and GM-CSF production in the supernatants was detected, respectively. The data are representative of more than three replicates.

T and NK cells are commonly employed for the expression of CARs (24). To evaluate the performance of VHH-CARs, we designed the other second-generation CAR constructs. The construct was optimized for CAR-NK cells, comprising a VHH domain directly linked to the hinge and transmembrane (TM) domains of CD8, followed by the co-stimulatory domain (CSD) of the OX40 and the CD3 zeta molecule in tandem (25, 26), and incorporating IL-15 and its receptor α-chain (15Rα) (27). (**Figure 4A**). Flow cytometry confirmed the CAR viral transduction efficiency, with positive rates exceeding 20% for both VHH-CAR-T and VHH-CAR NK cells (**Supplementary Figure S2A**). The observed poor transduction efficiency is likely related to the antibodies used for flow cytometry detection. Specifically, some clones may lose effective epitopes after humanization, leading to underestimation of the true transduction rate (28). To address potential inconsistencies in CAR positivity rates due to varying viral infection efficiencies, puromycin selection was used to ensure that only cells with successful CAR-Transduction were selected. This allowed for a more accurate comparison between the test and control CARs. Both cell types demonstrated significant cytotoxic effects against CLDN18.2-positive NUGC-4 cells *in vitro* (**Figures 4B, C**). However, the control naked NK cells exhibited strong nonspecific killing activity representing an intrinsic characteristic of NK cells, rendering them less suitable for evaluating VHH-CAR function(**Figure 4C**). Therefore, CAR-T cells were used for subsequent *in vitro* and *in vivo* experiments to validate the function of VHHs.

To evaluate the function of HM2-CAR-T cells, we performed an *in vitro* multi-round cytotoxicity assay against CLDN18.2-AsPC-1 and NUGC-4 cells. HM2 CAR-T cells exhibited a killing ability comparable to the scFv-based positive control, 8E5-2I -CAR-T cells (**Figures 4D, E**) (29). Moreover, cytokine secretion assays showed elevated levels of IFN-γ (**Figure 4F**), TNF-α (**Figure 4G**) and GM-CSF (**Figure 4H**) in the culture supernatants of CAR-T cells cocultured with CLDN18.2-AsPC cells. The cytokine response was indicative of a highly activated state. HM2-CAR-T cells exhibited the slightly higher production of pro-inflammatory cytokines compared to the 8E5-2I-CAR-T cells. These data collectively demonstrate that HM2-CAR-T cells possess robust effector functions *in vitro*, highlighting their potential and warranting further *in vivo* studies to assess their antitumor efficacy. e used quantitative cell-binding assays as a biologically relevant surrogate to estimate apparent affinity: serial dilutions of HM2-Fc were incubated with CLDN18.2-expressing cells, mean fluorescence intensity (MFI) was recorded by flow cytometry, and apparent equilibrium dissociation constants (apparent Kd) were estimated by non-linear curve fitting to a one-site saturation model. Cell-based binding provides an affinity estimate in the native membrane context and ensures the measured interaction is with the surface-exposed, physiologically relevant epitope. We have added the cell-binding curves and the derived apparent Kd values (**Figure 2**).

### HM2-CAR-T cells demonstrate potent antitumor activity *in vivo*

**Figure 5:**
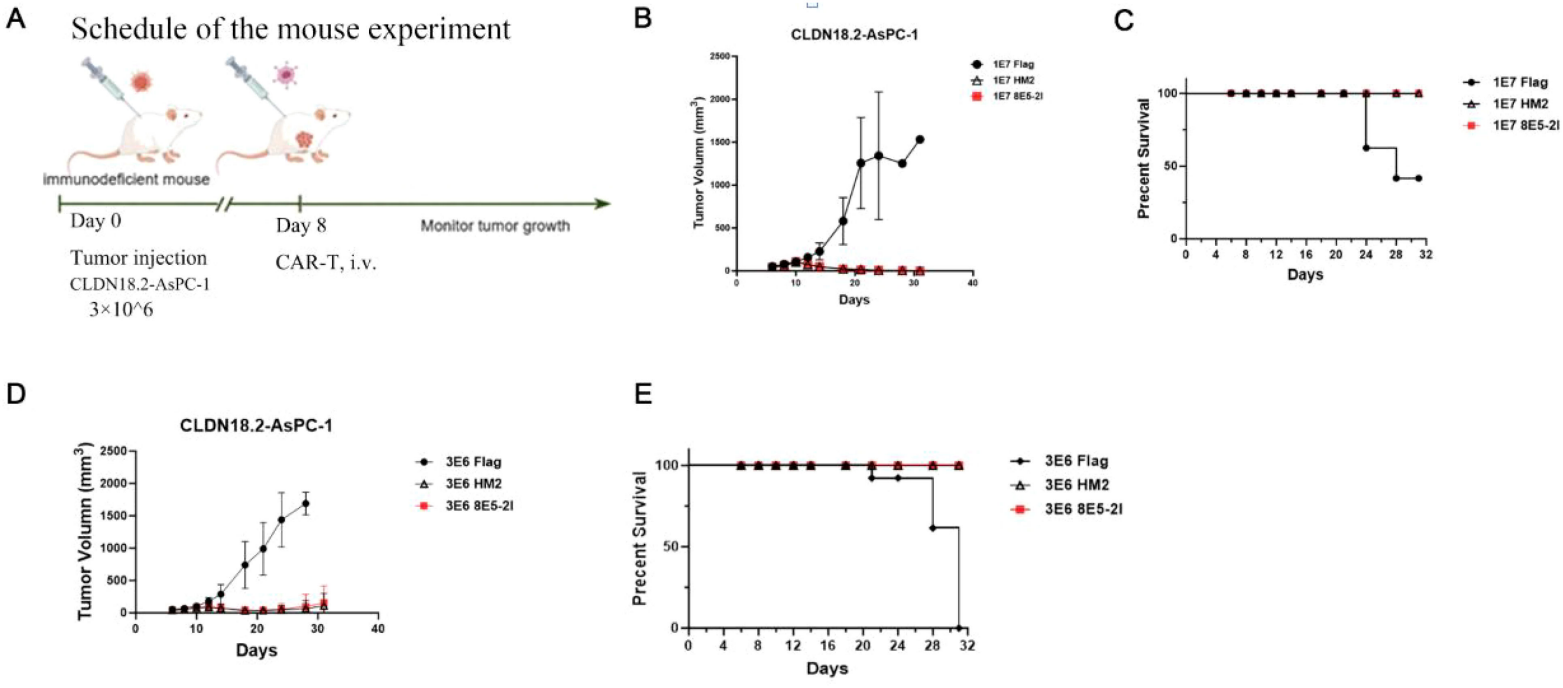
HM2-CAR-T cells exhibit high antitumor activity *in vivo*. **(A)** Experimental scheme of the mice experiment. **(B, D)** Tumor growth was assessed over time, and the results are expressed as the mean tumor volume (mm3 ± SD) with n=6 mice per group. **(C, E)** Survival curves were analyzed by using a log-rank test. .

Having demonstrated that HM2-CAR-T cells effectively killed CLDN18.2-expressing cells *in vitro*, we next evaluated their antitumor activity using the NPSG mouse model of PDAC. As shown in **Figure 5A**, NPSG mice were subcutaneously injected with 3×10^6^ CLDN18.2-AsPC-1 cells. Eight days later, the mice 1×10^7^ or 3×10^6^ HM2-CAR-T cells were injected via the tail vein. Tumor growth was significantly inhibited in the HM2-CAR-T cell-treated group compared to the FLAG control group (which had a DYKDDDDK tag instead of VHH domain). While tumors in FLAG control group progressed, HM2-CART cells effectively eliminated tumor burden. Notably, HM2-CAR-T cells and 8E5-2I -CAR-T cells exhibited comparable antitumor efficacy, with the tumor inhibition reaching 100% on day 17. (**Figure 5B**). As outlined in ethical guidance, mice with tumor volumes exceeding 2000 mm^3^ were euthanized. HM2-CAR-T cells significantly prolonged survival in tumor-bearing mice compared to the FLAG control group (**Figure 5C**). By 31 days post-tumor inoculation, all mice in the FLAG control group had succumbed to the disease, while mice treated with HM2-CAR-T cell displayed no detectable tumor burden (**Supplementary Figure S5A**). Furthermore, dose-reduction experiment (from 1×10^7^ to 3×10^6^ CAR-T cells) confirmed that HM2-CAR-T cells maintained robust antitumor efficacy comparable to 8E5-2I -CAR-T cells (**Figures 5D, E** and **Supplementary Figure S5B**). At 31 days post-treatment (study termination), all the mice were sacrificed. For safety evaluation, the vital organs (liver, spleen, lung, heart, kidney and stomach) from different mice were harvested immediately post- euthanasia. Organs were fixed in 4% formalin solution and histopathologically analyzed using hematoxylin and eosin staining. No toxicity-related abnormalities were detected (**Supplementary Figure S6**). These findings suggest that HM2-CAR-T cells demonstrated a favorable safety profile *in vivo.*, Of note, the safety profile persisted even in the presence of cross-reactivity with mouse CLDN18.2. Moreover, immunohistochemistry on gastric cancer tissues demonstrated that HM2-Fc detects CLDN18.2 in human tumors (**Supplementary Figure S4**). These findings support the clinical translational potential of HM2-CAR. Taken together, HM2-CAR-T cells demonstrated potent antitumor efficacy at both high and low doses, performing comparably to 8E5-2I CAR-T cells. These findings highlight HM2-CAR-T cells as a promising therapeutic option for CLDN18.2-expressing cancers.

## Discussion

VHHs from VHH phage libraries that specifically bind to CLDN18.2 epitope and constructed an anti-CLDN18.2 second-generation CAR-T cell using humanized VHH as the binding moiety were successfully isolated. The engineered CAR-T cells exhibited antibody-like specificity, enabling effective recognition of CLDN18.2-expressing cells. As a result, the HM2-based anti-CLDN18.2 CAR-T cells demonstrated significant cytokine secretion and cytotoxic activity *in vitro* and antitumor efficacy *in vivo* comparable to that of ScFv-based CAR-T cells highlighting their potential as a promising therapeutic alternative.

Previous studies have shown that VHH-based CAR-T cells possess antitumor activity comparable to scFv-based CAR-T cells (15, 26, 30). Nb derived from VHHs, offer unique advantages, including enhanced chemical stability and the ability to recognize specific epitopes (31). Given that the efficiency of viral packaging diminishes with increasing vector size (32), VHHs, with their compact gene fragments, present a distinct advantage, making viral vectors modified with them more amenable to efficient packaging. Moreover, VHHs display low immunogenicity due to their high sequence homology with human VH domains as demonstrated in various preclinical (33) and clinical trials (34). As such, CAR-T designs based on VHHs may hold greater clinical advantages (35). The findings herein support the advantages of using the VHH-CAR-T cells achieving comparable efficacy to scFv-based positive benchmark (29).

A systematic review concluded that high-affinity antigen-binding domain (ABDs) do not necessarily induce a high response rate of CAR-T cells therapy (36). The strength and durability of CAR-T cell function is not only influenced by antibody affinity but also determined by a combination of factors such as antigen density and CAR structure. Thus, multi-round cytotoxicity assay to assess the capacity of CAR-T cells for sustained killing of target cells represents a viable option. In this study, the candidate VHH 2-B08 was successfully selected for humanization due to its moderate affinity, robust multi-round cytotoxic function and high homology with human VH domains. As components of the innate immune system, NK cells exert cytotoxic effects against malignant cells (37). Even when genetically engineered to express CARs, NK cells retain their innate ability to recognize tumor cells through their endogenous receptors, providing an additional layer of antitumor reactivity distinct from CAR-T-mediated cytotoxicity (5, 38–43). CAR-NK cells have garnered considerable interest in recent years, due to the unique biological characteristics (44). Given the significant *in vivo* efficacy of HM2-CAR-T cells, we hypothesize that CAR-NK cells could yield comparable outcomes. Moreover, allogeneic NK cells have demonstrated a strong safety profile in cancer patients (41, 42). These attributes make CAR-NK cells, including HM2-CAR NK cells, promising candidates for targeting CLDN18.2-positive solid tumors. NK and T cells were both used to identify HM2-CARs with efficient anti-tumor functions. In the cytotoxicity assays against NUGC-4 cells, both HM2-CAR-T and HM2-CAR NK cells exerted robust killing capabilities. However, naked NK cells showed a background cytotoxic activity. Plausibly, this was specific to this particular cell line, and other target cell lines may be more compatible for screening CAR-NK cells. However, the non-specific killing observed in NUGC-4 cells raised concerns that naked NK cell activity could potentially confound *in vivo* validation results. Specifically, it would be difficult to distinguish whether the observed reduction in tumor size was related to the CAR or the inherent activity of the NK cells. Therefore, we prioritized T cells for further *in vivo* testing. Additionally, when NK cells were used to assess the potential of VHH-CARs, IL-15 was included in the CAR-NK cell system. We acknowledge that the experimental conditions involving different cytokines (IL-15 for NK cells and no cytokine for T cells) may have introduced variability such that IL-15 may contribute to some non-specific anti-tumor reactivity. However, IL-15 is crucial for maintaining NK cell activity and ensuring long-term efficacy *in vivo.*, Thus, it was deemed an important factor for optimizing NK cell function and improving the overall therapeutic potential of CAR-NK cells (42, 43). Although not specifically tested for graft-versus-host disease (GvHD), no significant weight loss or other signs of GvHD were observed during the study period. We speculated that, after tumor regression, the number of CAR-T cells decreased and did not persist at high levels for an extended period (5, 45) thereby minimizing the potential for GvHD development. The use of *in vivo* transduced cell lines that express CLDN18.2 at supra-physiological levels, introduces certain limitations. However, we believe that the data from NUGC-4 and the ICH analysis using human tumors provide valuable insights into the potential of HM2-CAR for clinical application.

Alpaca with purified human CLAUDIN 18.2 protein instead of challenged using CLDN18.2-expressing 293T cells, we would like to provide the following explanations. First, CLAUDIN 18.2 is a four-pass transmembrane protein, and purifying its extracellular domain in a functional form is quite challenging. The transmembrane nature of the protein makes it difficult to maintain its native conformation during purification processes. Therefore, we opted to use cells that express CLAUDIN 18.2 to ensure that the protein is presented in its natural state. Second, by using CLDN18.2-expressing 293T cells, we ensured that the protein was in its native conformation. This is crucial for generating antibodies that specifically target CLAUDIN 18.2 on the cell surface. We believe that our approach of using CLDN18.2-expressing 293T cells was the most appropriate method to achieve our research objectives. We hope this clarifies the rationale behind our experimental design. While this project had many strengths, it was not without limitations. We fully agree that Surface Plasmon Resonance (SPR) is a widely recognized technique for measuring the binding affinity of antibodies to antigens, providing valuable data such as the dissociation constant (KD). In fact, we have attempted to use SPR to measure the binding affinity of our antibodies to CLAUDIN 18.2. However, due to the challenges in expressing the extracellular domain of CLAUDIN 18.2 as a standalone protein, we utilized a Virus-Like Particle (VLP) structure of CLAUDIN 18.2 and immobilized it on the sensor chip via another anti-CLAUDIN 18.2 antibody. Unfortunately, despite our efforts, we were unable to detect normal binding of the antibodies to this form of CLAUDIN 18.2. Regarding the use of Flow Cytometry (FACS) to analyze antibody affinity, we would like to highlight its unique advantages in our context. FACS allows us to directly measure the binding of antibodies to cell surface proteins, which is highly relevant to our goal of screening antibodies that specifically target CLAUDIN 18.2 on the cell membrane. This method provides a more biologically relevant assessment of antibody binding, as it reflects the interaction in a cellular environment, which is the ultimate target for our antibodies. In summary, while we acknowledge the importance of SPR for measuring binding affinity, the challenges we encountered with the VLP structure of CLAUDIN 18.2 led us to rely on FACS, which offers a more direct and relevant measure of antibody binding to cell surface CLAUDIN 18.2. We acknowledge that alternative vector backbones and manufacturing optimizations can substantially increase transduction efficiency. To that end we have added a new paragraph to the Discussion that (a) acknowledges the limitation of the present transduction approach, (b) details practical options for process optimization (use of SFG or other gamma-retroviral backbones where appropriate, optimization of MOI, concentration of viral supernatant, Retronectin-coated plates, spinoculation, multiple rounds of transduction, and packaging system selection), and (c) commits to evaluating these strategies in future process development studies aimed at clinical translation. We also emphasize that despite the pre-selection transduction rate, the puromycin-enriched populations used for functional assays and the *in vivo* xenograft experiments show robust CAR expression and consistent anti-tumor activity, indicating that the observed biological effects are not attributable to a small, high-outlier subpopulation.

In conclusion, our results suggest the significant antitumor activity of VHH-based CLDN18.2-targeted CAR-T cells, with HM2-CAR-T cells emerging as a strong therapeutic candidate. However, further investigations are necessary to assess the safety and elucidate the detailed mechanism underlying their anti-tumor effects. Our findings provide compelling evidence supporting the clinical development of HM2-CAR-T cells as a promising immunotherapeutic strategy for CLDN18.2-expressing cancers.

## Data Availability

The datasets presented in this study can be found in online repositories. The names of the repository/repositories and accession number(s) can be found in the article/**Supplementary Material**.
