# Supplementary material for: VHH-based CAR-T cells targeting Claudin 18.2 show high efficacy in pancreatic cancer models
Source: Front Immunol. 2026 Jan 2;16:1638585. doi: 10.3389/fimmu.2025.1638585 (PMC12808354; doi:10.3389/fimmu.2025.1638585)
Supplement: Supplementary file 1 [file DataSheet1.pdf]

Supplemental Figures

Figure S1.

Supplemental information

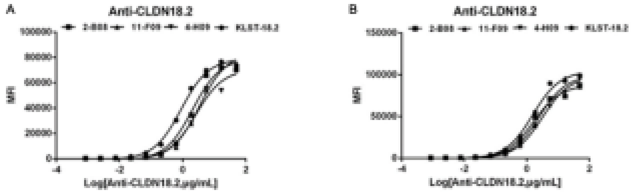

EC50	PC	2-B08	11-F09	4-H09
ug/ml	0.902	2.079	3.401	2.613
nM	11.2712	25.9875	42.5125	32.6625

EC50	PC	2-B08	11-F09	4-H09
ug/ml	1.558	1.636	2.479	3.18
nM	19.475	20.45	30.9875	39.75

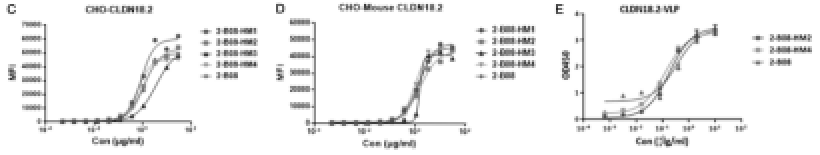

EC50	HM1	HM2	HM3	HM4	2-B08
ug/ml	0.878	1.167	3.645	0.638	1.113
nM	10.98	14.59	45.57	7.97	13.91

EC50	HM1	HM2	HM3	HM4	2-B08
ug/ml	1.362	1.59	1.538	0.992	1.407
nM	17.02	19.88	19.23	12.4	17.58

EC50	HM2	HM4	2-B08
ug/ml	0.182	0.124	0.344
nM	2.28	1.55	4.30

Figure S1 VHHs show high affinity to CLDN18.2 (A) and (B) The affinity of 2-B08, 11-F09, 4-H09, PC (positive control) to CHO-K1/18.2 and CHO-K1/Mouse 18.2 cells, respectively. The EC50 (concentration for 50% of maximal effect) is showed below the S-curve. (C), (D) and (E) The affinity of HM1, HM2, HM3, HM4, 2-B08 to CHO-K1/18.2 cells, CHO-K1/Mouse 18.2 cells and CLDN18.2-VLP, respectively. The EC50 is showed below the S-curve.

Figure S2.

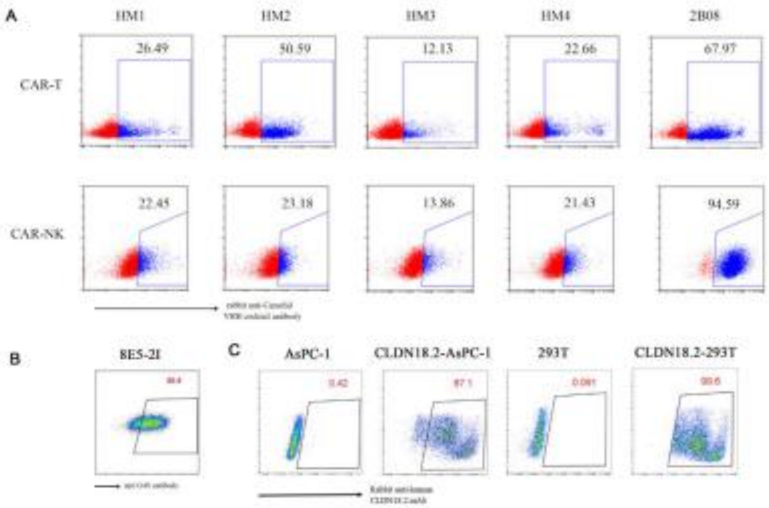

Figure S2 The transduction efficiency of CAR T and CAR NK cells, and CLDN18.2 expressing level ono target cells. (A) The transduction efficiency of VHH-CAR T and VHH-CAR NK cells. (B) The transduction efficiency of 8E5-2I-CAR T cells. (C) Representative flow cytometry plots of CLDN18.2 expression on different target cells.

Figure S3.

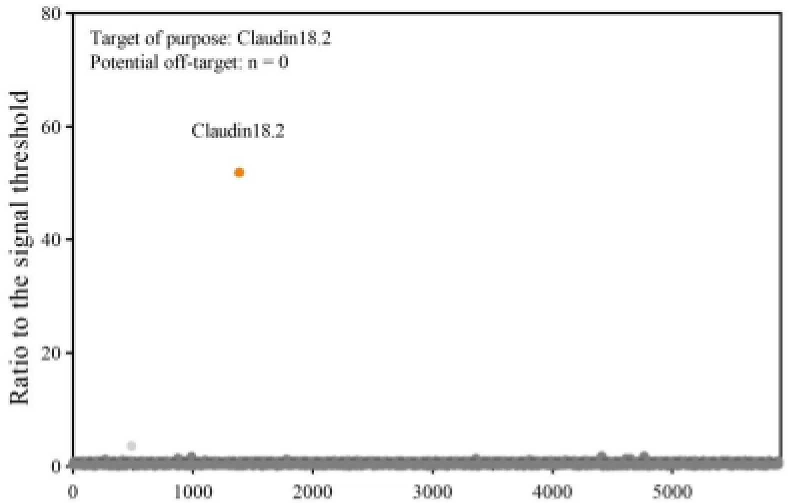

Figure S3 HM2 recognizes human CLDN18.2 in a specific manner. Membrane Protein Array screen of 5886 proteins for detecting potential off target binding of HM2. The HM2-Fc fusion protein utilizes the Fc domain from human IgG1, which allows detection using a secondary antibody.

Figure S4.

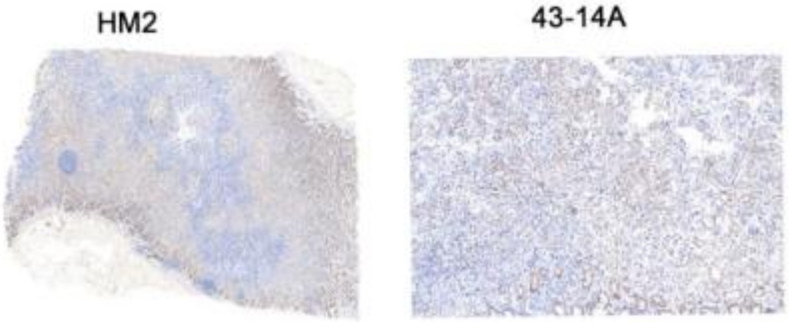

Figure S4 Representative IHC images of CLDN18.2 expression in gastric cancer. 43-14A (Cat# ab314690, abcam) represents positive control. The HM2-Fc fusion protein utilizes the Fc domain from human IgG1, which allows detection using a secondary antibody.

Figure S5.

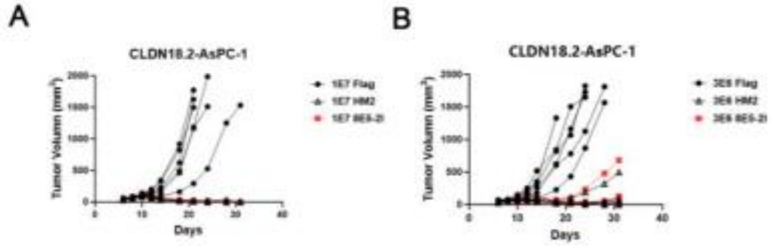

Figure S5. The tumor progression curves for individual mice.

Figure S6.

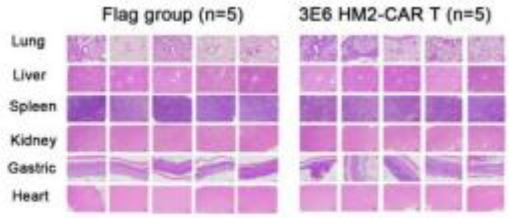

Figure S6 The histopathologically analysis by hematoxylin and eosin staining. All the mice were euthanized at the end of the study, and the vital organs were collected and subjected to pathological analysis.
