# Supplementary material for: VHH-based CAR-T cells targeting Claudin 18.2 show high efficacy in pancreatic cancer models
Source: Front Immunol. 2026 Jan 2;16:1638585. doi: 10.3389/fimmu.2025.1638585 (PMC12808354; doi:10.3389/fimmu.2025.1638585)
Supplement: Supplementary file 2 [file Table1.pdf]

Supplementary Table 1.

Tabel S1 The amino acid sequences of 2-B08, HM1, HM2, HM3 and HM4

Name	Sequence
**2-B08**	AVQLVESGGGLVQPGGSLRLSCAASGTIVLISTMAWYRQAPGKQREF<br>VAGISRGGTTNYADSVKGRFAISRDNTKNTMYLQMNSLQPDDTAVYY<br>CNADVSSVGWYNYWGQGTQVTVSS
**HM1**	EVQLVESGGGLVQPGGSLRLSCAASGTIVLISTMAWYRQAPGKGLEW<br>VAGISRGGTTNYADSVKGRFTISRDNSKNTMYLQMNSLRAEDTAVYY<br>CNADVSSVGWYNYWGQGTTVTVSS
**HM2**	EVQLVESGGGLVQPGGSLRLSCAASGTIVLISTMAWVRQAPGKGLEW<br>VAGISRGGTTNYADSVKGRFTISRDNSKNTMYLQMNSLRAEDTAVYY<br>CNADVSSVGWYNYWGQGTTVTVSS
**HM3**	EVQLVESGGGLIQPGGSLRLSCAASGTIVLISTMAWYRQAPGKQREFV<br>AGISRGGTTNYADSVKGRFAISRDNTKNTMYLQMNSLQPDDTAVYYC<br>NADVSSVGWYNYWGQGTQVTVSS
**HM4**	EVQLVESGGGLVQPGGSLRLSCAASGTIVLISTMAWYRQAPGKGLEW<br>VAGISRGGTTNYADSVKGRFTISRDNSKNTLYLQMNSLRAEDTAVYY<br>CNADVSSVGWYNYWGQGTTVTVSS
